# A ketogenic diet regulates microglial activation to treat drug addiction

**DOI:** 10.3389/fphar.2025.1462699

**Published:** 2025-01-23

**Authors:** Jie Ji, Yi Tang

**Affiliations:** ^1^ Department of Comprehensive (VIP) Inpatient Ward, Sichuan Clinical Research Center for Cancer, Sichuan Cancer Hospital & Institute, Sichuan Cancer Center, Affiliated Cancer Hospital of University of Electronic Science and Technology of China, Chengdu, China; ^2^ Department of Pharmacy, Sichuan Clinical Research Center for Cancer, Sichuan Cancer Hospital & Institute, Sichuan Cancer Center, Affiliated Cancer Hospital of University of Electronic Science and Technology of China, Chengdu, China

**Keywords:** ketogenic diet, microglia, inflammation, drug addiction, βhydroxybutyrate

## Abstract

Drug addiction is a chronic and potentially deadly disease that is considered a global health problem and describes the alteration of brain function by psychostimulant drugs through changes in the reward system. However, there is still no ideal strategy for the management of drug addiction. Previous studies have suggested that microglia are involved in events associated with neuroplasticity and memory, which are also related to drug addiction. Many studies have shown that psychoactive substances may act directly on immune cells, altering their function and inducing the production of various inflammatory mediators. In recent years, a ketogenic diet (KD) was shown to have therapeutic benefits as a dietary therapy for a variety of neurological disorders. With respect to drug addiction, studies have shown that a KD can alleviate glucose metabolism disorders caused by alcohol use disorders by increasing ketone metabolism, thereby reducing withdrawal symptoms. This finding indicates the potential of a KD as a treatment for drug addiction, since a KD may promote the transition of microglia to a predominantly anti-inflammatory state through several mechanisms. Here, we discuss recent research showing that a KD plays a variety of roles in controlling microglia-mediated inflammation, opening new treatment avenues to treat drug addiction. This succinct analysis offers evidence of the enormous potential of a KD to treat drug addiction through the inhibition of microglial activation.

## Introduction

Drug addiction is a chronic, recurrent condition that has recently gained recognition as a public health issue in numerous nations (Schottenfeld and O'Malley, 2016). The hallmarks of this illness are obsessively seeking and using a substance; an inability to control one’s intake of the substance; and a depressive mood, with little consideration for social, physical, or individual repercussions (Koob and Volkow, 2016).

Current studies have shown that signalling pathways involving dopamine receptors may be responsible for the adaptive cellular responses in the central nervous system (CNS) caused by prolonged exposure to psychoactive drugs. For example, D1 activation increases cAMP levels, which in turn activates extracellular signal-regulated kinase (ERK) and protein kinase A (PKA) (Andrianarivelo et al., 2019). The activation of these molecules leads to the activation of transcription factors such as delta FosB (ΔFosB), factors regulated by cAMP response element-binding protein (CREB), myocyte enhancer factor 2 (MEF2), and nuclear factor kappa B (NF-κB), which are related to changes in gene and protein expression (Nestler, 2012), receptor expression, neuronal excitability and cell morphology (Teague and Nestler, 2022). Psychostimulants can alter neuroplasticity-related signalling cascades, which are essential for the desire for psychostimulants and associated drug seeking and relapse (Krasnova et al., 2013).

Recently, it was demonstrated that microglia may regulate some of these molecular pathways. For example, microglia can change the shape and function of dopaminergic neurons by altering the expression of receptors and the levels of tyrosine hydroxylase (TH) and dopamine transporter (DAT); it has also been demonstrated the fluorescence intensity of DAT and TH in the ventral tegmental area (VTA) is decreased in male mice exposed to social stress compared with unhandled control male mice (Catale et al., 2022; Smith et al., 2020). Microglia also participate in glutamate-induced synaptic alterations by altering AMPA receptor expression, the AMPAR/NMDAR ratio, and glutamate release (Basilico et al., 2022; Ji et al., 2013). Finally, neurotransmission mediated by glutamate and dopamine also affects microglial activation (Yan et al., 2015), including in the context of psychostimulant abuse (Canedo et al., 2021). In this review, we found that the addiction-related behavioural alterations caused by binge methamphetamine exposure are mediated by astrocyte‒microglia crosstalk, in which the release of glutamate from astrocytes in a TNF/IP3 receptor (IP3R)/SNARE-dependent manner leads to microglial activation, neuroinflammation, and ultimately changes in addictive behaviour in mice (Canedo et al., 2021).

Encouragingly, multiple studies have revealed a strong correlation between diet-induced ketosis and the primarily anti-inflammatory polarization of microglia in animals (Fedorovich et al., 2018; Ghosh et al., 2018). Moreover, several researchers have reported that nutritional ketosis results in the inhibition of molecules such as mitogen-activated protein kinase (MAPK), p38, NF-κB and the nucleotide-binding, leucine-rich repeat, pyrin-domain-containing 3 (NLRP3) inflammasome (Guo et al., 2018; Trotta et al., 2019) in addition to an increase in the synthesis of substances such as peroxisome proliferator-activated receptor (PPAR) and interleukin (IL)-10 (Newman et al., 2017). These changes may favour a switch to predominantly anti-inflammatory/neurorestorative microglial polarization (Xu et al., 2018; Yang et al., 2018). Therefore, these findings suggest that diet-induced ketosis may have pleiotropic effects on important mediators of microglial function. If this is also true in human patients, new avenues for treating neurological disorders could be explored.

A ketogenic diet (KD) is a diet with a reduced proportion of carbohydrates and an increased proportion of fat (Veech, 2004). A KD is characterized by adequate energy and protein intake and the restriction of carbohydrates, typically to less than 30–50 g/day (Trimboli et al., 2020). In a high-fat diet, calories from fat typically constitute 30%–75% of the total daily caloric intake. However, some nutritional protocols may aim for an even higher percentage of calories from fat, reaching up to 90%. High-fat diets are usually characterized by excessive intake of saturated fatty acids and calories, which can lead to an increase in health problems, particularly heart disease, weight gain, and obesity (Han et al., 2023).

A KD induces a state of ketosis that is similar to that caused by fasting. Normally, carbohydrates are converted to glucose, which the brain uses as its primary energy source. However, when no carbohydrates are available, the body uses other energy sources, such as acetyl coenzyme A (ac-CoA), which produces excess ketone molecules, including acetoacetate (ACA), β-hydroxybutyrate (BHB), and acetone, through a process called ketogenesis (Fukao et al., 2004). Unlike pathological ketoacidosis, ketosis is a normal process, as ketone bodies can be utilized effectively without reaching dangerous levels (Paoli et al., 2013). In the past, a KD was widely and successfully used to treat epileptic disorders (Neal et al., 2008). According to recent studies, a KD may also be beneficial for treating alcohol or drug addiction (Li et al., 2022; Wiers et al., 2021). For example, research has demonstrated that a KD with a ratio of fats to carbohydrates and proteins of approximately 6:1 (93% fat, 2% carbohydrates and 5% protein; 5 weeks) interferes with the sensitization of ambulatory responses in cocaine-treated animals and reduces the stereotyped responses elicited by cocaine in rats (Martinez et al., 2019);. In clinical studies, a KD (80% fat, 15% protein, and 5% carbohydrates; 3 weeks) reduced alcohol craving and withdrawal symptoms in patients with alcohol use disorders. In addition, BHB, a KD metabolite and the most abundant ketone body, can β-hydroxybutyrylate CaMKII-α, resulting in significant inhibition of T286 autophosphorylation and decreased CaMKII activity, which plays a critical role in mediating the effect of KD consumption in reducing cocaine reinstatement (Li et al., 2022).

## The biochemistry of ketogenesis

Under physiological conditions, fatty acid oxidation produces acetyl-CoA, which enters the tricarboxylic acid (TCA) cycle and reacts chemically with oxaloacetate to form citrate. However, under the metabolic conditions induced by a KD, oxaloacetate is released from mitochondria and used in gluconeogenesis (Elamin et al., 2017). Under these conditions, the amount of oxaloacetate in the mitochondrial environment is far less than the amount of acetyl-CoA produced, and oxaloacetate undergoes a series of condensation processes that are characteristic of ketogenesis (Kim et al., 2010). First, acetoacetyl-CoA is generated by the combination of two acetyl-CoA molecules. HMG-CoA synthase 2 facilitates a functionally irreversible and rate-limiting interaction between acetoacetyl-CoA and another acetyl-CoA molecule to generate HMG-CoA (Hyatt et al., 2016). After HMG-CoA is produced, it dissociates to form ACA, which is then further reduced to BHB via a process facilitated by BHB dehydrogenase in which nicotinamide adenine dinucleotide (NAD)/NADH acts as a hydrogen donor (Bentourkia et al., 2009). Notably, BHB is the major ketone body (KB) because the level of BHB in the circulation and tissues is significantly greater than the level of ACA (Pifferi et al., 2008).

**FIGURE 1:**
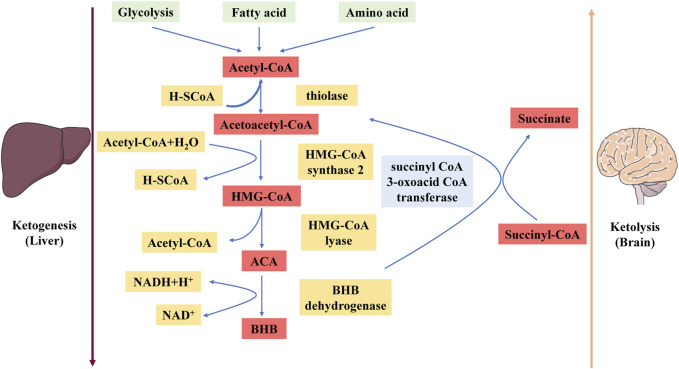
Schematic diagram depicting the ketogenesis and ketolysis reactions. ACA, acetoacetate; BHB, β-hydroxybutyrate. Under physiological conditions, acetyl-CoA produced by fatty acid oxidation enters the tricarboxylic acid (TCA) cycle and subsequently engages in a chemical reaction with oxaloacetate to produce citrate. The level of synthesized acetyl-CoA greatly exceeds the amount of oxaloacetate in the mitochondrial environment, and acetyl-CoA engages in a series of condensation reactions, which are hallmarks of ketogenesis. First, two acetyl-CoA molecules combine to produce acetoacetyl-CoA. This molecule reacts with another acetyl-CoA molecule to form HMG-CoA *via* a functionally irreversible and rate-limiting reaction facilitated by HMG-CoA synthase 2. Once formed, this compound dissociates to form the KB acetoacetate (ACA), which is further reduced to BHB via a reaction facilitated by BHB dehydrogenase in which NAD^+^/NADH acts as the hydrogen donor. BHB is released into the circulation from the liver and ultimately enters the brain.

The liver releases BHB and ACA into the bloodstream, where they are subsequently taken up by the heart, brain, skeletal muscle, and other tissues with high metabolic needs (Kim et al., 2010). Once BHB reaches these tissues, BHB dehydrogenase converts BHB to ACA, which functions as a major regulator of the mitochondrial NAD^+^/NADH ratio (Pifferi et al., 2011). The enzyme succinyl-CoA:3-oxoacid CoA transferase then catalyses the hydrolysis of ACA to produce acetoacetyl-CoA and succinate, and thiolase cleaves acetoacetyl-CoA to yield acetyl-CoA. Acetyl-CoA and succinate serve as substrates for the TCA cycle and complex II of the electron transfer chain (ETC.), respectively (Roy et al., 2012) (Figure 1). This mechanism may explain the increased succinate dehydrogenase activity in rodents fed a KD for extended periods (Hashim and VanItallie, 2014). The effects of a KD can be replicated with KB supplements, and while not widely accepted, there is evidence that the normal ability of the liver to produce KBs may be impeded by KB supplementation (Hugo et al., 2012).

## Role of microglia in neural function and CNS homeostasis

Microglia surveil and quickly react to foreign substances under physiological conditions, actively observing and controlling alterations in neuronal activity (Schafer et al., 2012). Microglia take on this role because they briefly come into contact with synapses and extrasynaptic regions through highly coordinated movement (Salter and Beggs, 2014), and they express homologous receptors for neurotransmitters such as glutamate and gamma-aminobutyric acid (GABA) as well as receptors for a variety of neuronal mediators, such as CD200 and fractalkine (CX3CL1), on their surfaces (Koizumi et al., 2013). Additionally, microglia express receptors for purinergic neurotransmitters such as adenosine triphosphate (ATP), serotonin, and acetylcholine (Schafer et al., 2012). An overwhelming body of research suggests that ATP, which is generated by neurons, is a key regulator and the most effective inducer of microglial activity (Orr et al., 2009).

Numerous research groups have reported that microglia may control GABAergic and glutamatergic neurotransmission (Pascual et al., 2012). ATP, which binds to cognate receptors on astrocytes and increases glutamatergic activity by upregulating metabotropic receptor 5, is the most likely regulator of glutamatergic neurotransmission (Pascual et al., 2012). The ability of microglia to regulate GABAergic transmission is particularly significant under pathological conditions. This regulatory process involves the release of ATP from injured neurons, which elicits the release of brain-derived neurotrophic factor (BDNF) from activated microglia. The released BDNF has the potential to reverse the direction of GABAergic neurotransmission (Ferrini and De Koninck, 2013).

## Responses of microglia to cocaine

**FIGURE 2:**
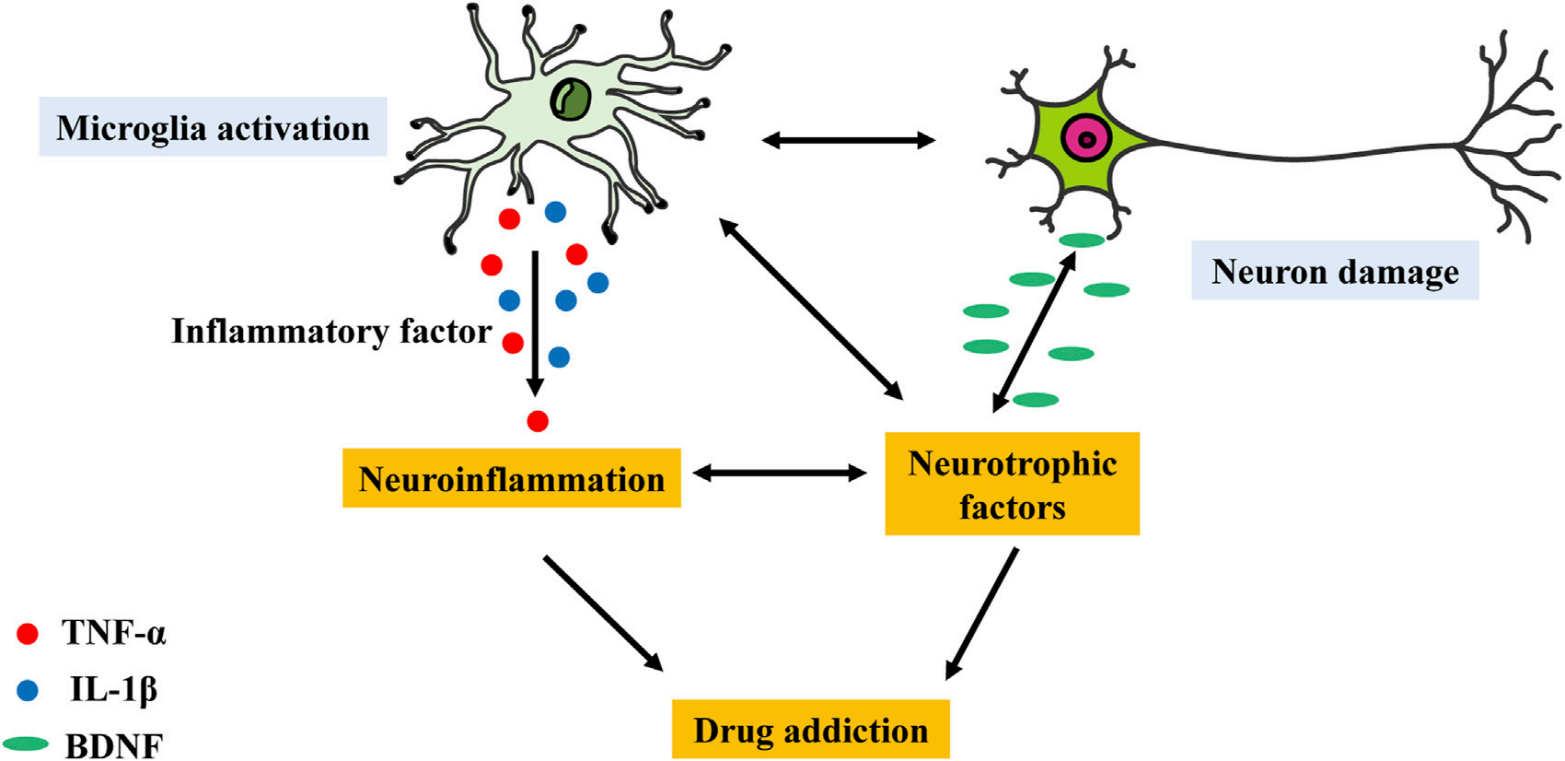
Possible effects through which the microglial activation–neuronal damage interaction may lead to neuroinflammation and further mediate drug addiction. Disruption of the interaction between microglial activation and neurons via inflammatory factors or neurotrophic factors may lead to neuroinflammation and drug addiction.

Although changes in the glutamatergic and dopaminergic systems are thought to be the primary neurobiological mechanisms governing motivated behaviour, it is widely recognized that psychoactive substances can also modify the glutamatergic, serotonergic, and GABAergic neurotransmitter systems as well as the levels of various molecules, such as cytokines and neurotrophic factors (Doggui et al., 2021). Furthermore, microglia can respond to neurochemical alterations caused by psychoactive substances because they express ion channels and neurotransmitter receptors, which are likewise expressed by neurons (Figure 2). Notably, microglial activation has been demonstrated to alter the reward system (Taylor et al., 2015), mostly due to the high susceptibility of dopaminergic neurons to neuroinflammatory signals (Douma and de Kloet, 2020).

### Microglia and cocaine

The crucial role of microglia in the development and maturation of synapses is widely acknowledged. Drugs of abuse impair synaptic and neuronal function over time. Thus, increasing amounts of data point to a possible link between drug addiction and glial function abnormalities (Kierdorf and Prinz, 2017; Schmidt and Engel, 2021). In particular, the abuse of opioids, alcohol, and psychostimulants is thought to be influenced by the glial and neuroimmune systems. It is hypothesized that substances of abuse activate microglia by stimulating innate immune receptors, causing the release of cytokines and chemokines, which have an impact on neuronal function (Schmidt and Engel, 2021). Moreover, innate immune receptor stimulation may cause synaptic remodelling directly (Schmidt and Engel, 2021). A theory known as the “xenobiotic hypothesis” has emerged recently. This hypothesis suggests that because substances of abuse are exogenous, they are viewed as foreign “invaders” that activate the immune system’s defence mechanism. Microglia, the main resident immune cells in the brain, act as the first line of defence. However, repeated and continuous administration of substances of abuse results in hyperactivation of microglia and a neuroinflammatory state, which can further exacerbate drug addiction by altering neuronal function (Deng et al., 2020). Microglia play an important role in drug addiction; a recent study showing that microglia are required for synaptic alterations during cocaine withdrawal lends further credence to this speculation (Cadet and Bisagno, 2014). More research on microglial overactivation might improve our knowledge of the mechanism of drug addiction and possibly lead to the development of innovative treatments.

Preclinical research has shown that cocaine upregulates the expression of the traditional microglial marker ionized calcium-binding adaptor molecule-1 (Iba-1) in the hippocampus, frontal cortex, and nucleus accumbens (NAc) (Costa et al., 2013; Jarvis et al., 2019), as well as in sections of the mouse and rat brain (Chivero et al., 2020). Furthermore, cocaine upregulates the expression of CD11 in the striatum, cortical areas, and VTA (Chivero et al., 2021). Interestingly, research has shown that acute cocaine injection elevates CD11 expression only in animals with a history of cocaine self-administration (Brown et al., 2018). Moreover, cocaine increases the expression of CD68, a transmembrane glycoprotein expressed by microglia, indicating increased phagocytic activity (da Silva et al., 2021). Cocaine affects the number and shape of microglia in addition to their protein expression. For example, research has shown that after cocaine is used for 7 days in a row, more cells express allograft inflammatory factor 1 (AIF1) (Thangaraj et al., 2020). Additionally, previous studies have shown that cocaine exposure decreases the number of microglial branches and increases the size of microglial cell bodies (Burkovetskaya et al., 2020; da Silva et al., 2021). In other words, cocaine causes morphologic changes that could be linked to heightened microglial activation.

Finally, the behavioural and molecular effects of psychostimulants can be altered by microglial activity (Linker et al., 2020). The inhibition of microglia by minocycline, a common tetracycline antibiotic, reverses the effects of cocaine on behaviour, conditioned place preference (CPP), and dopamine release (Northcutt et al., 2015). Moreover, minocycline also modifies cocaine reward after exposure to morphine and nicotine (Taylor et al., 2016). Furthermore, the glial modulator and phosphodiesterase 4 (PDE4) inhibitor ibudilast ameliorates cocaine addiction in both humans and animals (Mu et al., 2021), and the colony stimulating factor-1 (CSF-1) inhibitor PLX3397 also reduces cocaine-induced behavioural changes by depleting microglia (Linker et al., 2020).

There have been few clinical studies on the effects of cocaine on microglia in humans, and those that exist have generated controversial results. The only study that was performed to assess cocaine-induced microglial activation in a clinical population exploited the binding of the PET radioligand [11C]PBR28 to TSPO (Narendran et al., 2014). However, the authors failed to demonstrate an alteration in [11C]PBR28 binding to TSPO in abstinent (for a minimum of 14 days) patients who met the DSM-IV criteria for cocaine dependence compared with healthy controls (Narendran et al., 2014).

## Positive effects of induced ketosis on anti-inflammatory microglial polarization

### Increased NAD^+^ production and GPR109A receptor activation

Numerous studies have confirmed that, after prolonged ketosis, NAD^+^ levels are increased in the CNS of animals (Xin et al., 2018). Other researchers have reported the inhibition of glycolysis in the brains of study participants (Courchesne-Loyer et al., 2017). These findings may have therapeutic implications since the elevation of NAD^+^ levels and simultaneous reduction in NADH levels, along with the inhibition of glycolysis, may cause the anti-inflammatory effects of a ketotic state on microglia in the brain. An increase in the NAD/NADH ratio has an important effect, as it alters the activity of the transcriptional inhibitor C-terminal-binding protein (CtBP), which binds to the acetyltransferase p300 in microglia and other myeloid lineage cells to regulate the transcription of NF-κB and the expression of other proinflammatory genes. However, this hypothesis needs to be empirically confirmed (Shen et al., 2017). One of the defining characteristics of proinflammatory microglial polarization is increased glycolysis, which increases NADH levels and prevents CtBP from dimerizing, eliminating its ability to function as a transcriptional repressor. The opposite is true for decreased glycolysis and increased oxidative phosphorylation, which increase NAD^+^ levels (Ghosh et al., 2018; Shen et al., 2017). Notably, an increase in NAD^+^ levels in the brain due to diet-induced ketosis may have a beneficial anti-inflammatory effect through the binding of CtBP to the promoter regions of genes that promote inflammation in microglia. Moreover, research suggests that a more direct way to produce the same effect is through the binding of KBs, particularly BHB (Pinto et al., 2018). Furthermore, because KBs promote the primarily anti-inflammatory polarization of microglia, they may represent potential therapeutic options.

According to previous research, BHB inhibits the generation of COX-2 and iNOS by activating microglia both *in vivo* and *in vitro*, partly through the activation of G protein-coupled receptor 109A (GPR109A) (Fu et al., 2015). This receptor decreases the degradation of IkBa and prevents NF-κB from translocating to the nucleus, which allows the NF-κB to induce the transcription of inflammatory molecules (Fu et al., 2015). Studies have also shown that rats that have ingested or been injected with BHB have lower NLRP3 activity in microglia and other cells of the CNS. This decrease in activity could contribute independently to a reduction in neuroinflammation by lowering IL-1 and IL-18 levels (Yamanashi et al., 2017). Additionally, this effect may be partially mediated by binding to GPR109A and the consequent suppression of NLRP3 assembly due to endoplasmic reticulum (ER) stress (Youm et al., 2015). Although clearly important, this mechanism is not the only one through which BHB administration may decrease ER stress and, subsequently, the *in vivo* activation of the inflammasome. For example, BHB can reduce ER stress by blocking mitochondrial fragmentation mediated by dynamin-related protein 1 (DRP-1) and stimulating AMP-activated protein kinase (AMPK) (Bae et al., 2016). From the perspective of anxiety and depression treatment, the potential of diet-induced ketosis to inhibit NLRP3 activity in the brain is interesting because there is increasing evidence that stress-mediated activation of this inflammasome precipitates or exacerbates anxiety and depression symptoms, whereas its inhibition results in their amelioration or, in certain cases, termination (Iwata et al., 2016).

### Suppression of histone deacetylase

Nuclear factor erythroid 2-related factor 2 (Nrf-2) in the brain is also upregulated by ketosis (Milder et al., 2010). Although the underlying mechanisms are unknown, this change appears to be directly caused by BHB (Izuta et al., 2018) and probably occurs via histone deacetylase inhibition (Cai et al., 2015). In animal models of various neurological diseases or traumatic brain injury, Nrf-2 upregulation has been shown to be positively correlated with the predominantly anti-inflammatory polarization of microglia, with concomitant decreases in iNOS and IL-6 production as well as the induction of anti-inflammatory M2 polarization *in vivo*. Studies have explored the potential of Nrf-2 upregulation as a therapeutic approach for reducing neuroinflammation (Li et al., 2018). Although the mechanisms underlying these beneficial effects of Nrf-2 upregulation in ameliorating microglial activity and neuroinflammation involve the inhibition of inflammatory factor secretion and NLRP3 activity, they are complementary to the mechanisms underlying the effects of KD consumption and BHB administration. However, they are sufficiently different to suggest a level of synergy that would not be possible by modifying a single biochemical pathway (Ahmed et al., 2017). For example, the anti-inflammatory effect of Nrf-2 activity results from conserved cross-talk between Nrf-2 and NLRP3 *in vivo*, which is facilitated by a sophisticated mechanism that works antagonistically with the Rho family kinase RAK to limit the development of inflammation and oxidative stress (Cuadrado et al., 2014).

## Conclusion and outlook

In this article, we summarize the intriguing potential of a KD to treat drug addiction through the modification of microglial activation. Interestingly, the possible therapeutic benefits of KD consumption and other strategies for fostering ketosis for CNS diseases have not received much attention in the field of neuropsychiatry. The neurobiological mechanisms of various mental disorders, especially drug addiction, may be significantly influenced by primarily proinflammatory microglial polarization, as indicated by accumulating evidence from preclinical, *postmortem*, and *in vivo* human studies. A KD was shown to be beneficial in various neurological disorders, which suggests that its potential to treat drug addiction cannot be disregarded. Decreasing microglial activation *via* consumption of a KD might be beneficial for numerous illnesses caused by drug addiction.

KD consumption may be promoted recovery from drug addiction through various effects. However, some researchers have shown concerns regarding the consumption of a KD in individuals neurological diseases, such as decreased appetite, increased risk of malnutrition, and several adverse effects (Włodarek, 2019). The common adverse effects of KD consumption include metabolic abnormalities, gastrointestinal symptoms, kidney stones, and slow growth in children (Kossoff et al., 2009). However, most of these adverse effects were observed in children. Therefore, a KD should be applied with caution in people with drug addiction, as this particular population often suffers from multisystem disorders such as increased risk of malnutrition (Jeynes and Gibson, 2017) and gastrointestinal symptoms (Su et al., 2020). Finally, further preclinical studies and randomized controlled clinical trials are needed to optimize KD strategies, such as the timing of intervention and nutrient composition, and assess the suitability, effectiveness, and safety of a KD in the treatment of drug addiction.
